# Comprehensive deep learning-based assessment of living liver donor CT angiography: from vascular segmentation to volumetric analysis

**DOI:** 10.1097/JS9.0000000000001829

**Published:** 2024-06-13

**Authors:** Namkee Oh, Jae-Hun Kim, Jinsoo Rhu, Woo Kyoung Jeong, Gyu-Seong Choi, Jongman Kim, Jae-Won Joh

**Affiliations:** a Department of Surgery, Samsung Medical Center, Sungkyunkwan University School of Medicine, Seoul; b Department of Radiology, Samsung Medical Center, Sungkyunkwan University School of Medicine, Seoul, Korea

**Keywords:** deep learning, image processing, liver transplantation

## Abstract

**Background:**

Precise preoperative assessment of liver vasculature and volume in living donor liver transplantation is essential for donor safety and recipient surgery. Traditional manual segmentation methods are being supplemented by deep learning (DL) models, which may offer more consistent and efficient volumetric evaluations.

**Methods:**

This study analyzed living liver donors from Samsung Medical Center using preoperative CT angiography data between April 2022 and February 2023. A DL-based 3D residual U-Net model was developed and trained on segmented CT images to calculate the liver volume and segment vasculature, with its performance compared to traditional manual segmentation by surgeons and actual graft weight.

**Results:**

The DL model achieved high concordance with manual methods, exhibiting Dice Similarity Coefficients of 0.94±0.01 for the right lobe and 0.91±0.02 for the left lobe. The liver volume estimates by DL model closely matched those of surgeons, with a mean discrepancy of 9.18 ml, and correlated more strongly with actual graft weights (R-squared value of 0.76 compared to 0.68 for surgeons).

**Conclusion:**

The DL model demonstrates potential as a reliable tool for enhancing preoperative planning in liver transplantation, offering consistency and efficiency in volumetric assessment. Further validation is required to establish its generalizability across various clinical settings and imaging protocols.

## Introduction

Highlights

- The study highlights significant enhancements in liver transplantation planning through 3D modeling software, which offers improved accuracy and detailed visualization of liver volumes and vascular structures.
- The deep learning model, a 3D residual U-Net, achieved high accuracy in segmenting liver structures, demonstrating Dice Similarity Coefficients of 0.94 for the right lobe, 0.91 for the left lobe, and robust performance across individual liver sections.
- The deep learning model demonstrated strong correlation between estimated and actual liver volumes, suggesting its potential to replace manual volumetric assessments with more reliable automated estimations.

In living donor liver transplantation, assessing the vascular structure of the donor through preoperative computerized tomographic angiography (CTA) is crucial for the detailed screening of donor eligibility^1,2^. The subsequent volumetric assessment based on these CTA findings influences both donor safety and recipient outcomes. The future remnant liver volume of donor should be at least 30% to ensure donor safety^3,4^, while the graft volume also achieve a graft-recipient weight ratio of more than 0.6–0.8% to fulfill the recipient’s metabolic demands, avoiding small for size syndrome^5,6^.

Traditionally, preoperative volume measurements required manual segmentation by surgeons, who would trace the resection plane in accordance with the major hepatic vessels depicted in CT scans^7–9^. Nowadays, with the advent of 3D modeling software and its commercial availability, this process has been significantly refined^10^. At our institution, we maintain the use of traditional volumetric evaluations conducted by surgeons, yet we have also integrated 3D modeling software for measuring liver volume since 2022. This process offers a comprehensive analysis of volume and detailed visualization of the portal and hepatic veins (HV), enhancing our intraoperative navigation capabilities. While the clinical utility of these 3D models expires after surgery, the data derived from the manual segmentation becomes a valuable resource. This repository of segmented images has the potential to train artificial intelligence systems by leveraging deep learning (DL) to automate the segmentation process that was previously performed manually^11,12^.

In this study, we present the outcomes of a DL model designed for measuring sectional liver volume and autosegmentation of vascular structures from CTA of living liver donors. We compared these automated results with those obtained from manual segmentation and the actual measured liver weight.

## Methods

### Patient

This investigation included living liver donors from Samsung Medical Center, Seoul, Korea, between April 2022 and February 2023. Each donor underwent a comprehensive evaluation for liver donation candidacy, including CTA. Demographic information such as age, sex, and BMI were retrieved in a de-identified state from the Clinical Data Warehouse DARWIN-C of Samsung Medical Center. This study was approved by the institutional review board of Samsung Medical Center (SMC-2024-03-107) and the need for informed consent was waived by the IRB due to the retrospective and observational nature of the study. It was carried out in accordance with the principles of the Declaration of Helsinki.

### 3D modeling process

3D modeling, facilitated by CTA for all donors, was prospectively performed to better understand donor anatomy preoperatively. Hepatic parenchyma and vascular structures, including the portal vein (PV), HV, and inferior vena cava, were manually segmented by two independent biomedical engineers using Mimics Medical Software (Materialise). These preliminary segmentation maps were subsequently reviewed and, if necessary, revised by a board-certified abdominal radiologist and liver surgeons. Discrepancies in initial annotations were corrected under the supervision of experienced physicians. The liver parenchyma was categorized into the right posterior section (RPS), right anterior section (RAS), left medial section (LMS), left lateral section (LLS), and Spigelian lobe (SL), based on PV distribution.

### Volumetric assessment by surgeon and 3D software

The traditional method for liver volume assessment involved measuring the product of the area of the right and left hemiliver based on the right and left PV territory and the border of middle HV and the slice interval of CT images. Following measurements on all relevant CT images, these areas were integrated to estimate the liver volumes, a process performed by liver surgeons for all donors. The 3D model’s liver volume was computed with Mimics Medical Software. The actual graft weight was determined by its postperfusion weight following procurement surgery.

### Segmentation model

Our study employed a 3D residual U-Net model, which uses an encoding process to extract contextual features and a decoding process to reconstruct the segmented image. The model was trained to minimize a specific loss function, fine-tuning the network parameters for hierarchical feature extraction. It incorporates convolutional residual operations in encoding and utilizes deconvolutional feature maps with skip connections for reconstructing multitarget segmented images during decoding.

### Implementation

We implemented the 3D residual U-Net model in TensorFlow 1.14 on a workstation equipped with four NVIDIA TITAN XP 16GB GPUs. Image preprocessing involved cropping, resizing, and normalization. Data augmentation included 3D rotation, scaling, random flipping, and cropping. The dice similarity coefficient (DSC) loss function guided the training, with the Adam optimizer set to a 0.0001 learning rate. Training spanned 1000 epochs with a batch size of four. For testing, full CTA scans underwent preprocessing before being inputted into the network.

### Evaluation and statistical analysis

The model’s training incorporated 90% of patients, with validation on the remaining 10%. The performance of the DL model to segment liver parenchyma, HV, and PV was compared with manually delineated ground truth. As a quantitative measure, DSC was used to quantify the segmentation performance of the DL model. For assessing the DSC coefficient, 3D slicer was used for calculation. Bland–Altman analysis examined volumetric differences between the surgeon’s estimate and the model’s output. Correlation of estimated and actual liver graft weight measurements was analyzed using Pearsons correlation. The Wilcoxon signed rank test was used to compare the estimated liver volume measurements to the actual graft weight. Continuous variables were presented as mean±SD and analyzed using the independent *t*-test or Mann–Whitney test, as appropriate. Categorical data were presented as numbers and percentages and analyzed using the *χ* ^2^ or Fisher’s exact test. All statistical analyses were performed using Python 3.8 (Python Software Foundation).

### Data availability

Data generated or analyzed during the study are available from the corresponding author by request.

## Results

### Donor characteristics

In this study, 114 patients were evaluated, with 103 forming the training set and 11 comprising the validation set. There were no statistical differences in sex, age, or BMI of the donors between the two groups (Table 1). Type I PV was the most common in both groups, occurring in 98 patients, followed by type 2 in 9 patients, and type 3 in 7 patients, with no difference in proportion observed between the groups. Of these patients, 104 donors were prepared for a right graft, 3 for a left graft, and 7 donors were not proceeded with for donation. Regarding the estimated liver volume measured by surgeon, the mean±SD total liver volume was 1269.3±246.0 ml in the training set and 1207.0±281.5 ml in the validation set (*P*=0.494), showing no statistical difference. For the right liver only, the volumes were 794.0±168.7 ml in the training set and 788.8±162.5 ml in the validation set (*P*=0.921). The ratio of the right liver volume was 62.5±4.9% in the training set and 65.8±4.3% in the validation set (*P*=0.034).

**Table 1:** Donor characteristics.

	Train set (*n*=103)	Test set (*n*=11)	*P*
Age (mean [SD])	37.8 [12.4]	41.9 [12.7]	0.35
Male sex (%)	52 (50.5)	4 (36.4)	0.386
BMI (mean [SD])	24.0 [3.3]	24.0 [3.0]	0.967
PV type (%)			
I	88 (85.4)	10 (90.9)	1
II	8 (7.8)	1 (9.1)	
III	7 (6.8)	0 (0.0)	
Estimated liver volume by manual segmentation (ml, mean [SD])			
Whole liver	1269.3±246.0	1207.0±281.5	0.494
Right hemiliver	794.0±168.7	788.8±162.5	0.921
Left hemiliver	437.4±185.5	413.8±120.7	0.571
Right posterior section	332.2±94.3	335.4±93.2	0.915
Right anterior section	461.8±133.1	453.3±98.5	0.799
Left medial section	166.8±53.0	162.2±43.6	0.749
Left lateral section	238.2±164.4	226.3±75.4	0.674
Spigelian lobe	32.4±28.4	25.3±10.1	0.096
Volume ratio (%, mean)			
Right hemiliver	62.5±4.9	65.8±4.3	0.034
Left hemiliver	34.5±14.7	34.0±4.0	0.798
Right posterior section	26.3±6.3	27.7±3.8	0.29
Right anterior section	36.2±6.2	38.1±6.3	0.377
Left medial section	13.0±2.8	13.5±2.3	0.565
Left lateral section	18.9±14.3	18.4±2.6	0.756
Spigelian lobe	2.5±1.8	2.1±0.6	0.089

PV, portal vein.

### Quantitative evaluation for segmentation of parenchyma, PV, and HV

**Figure 1:**
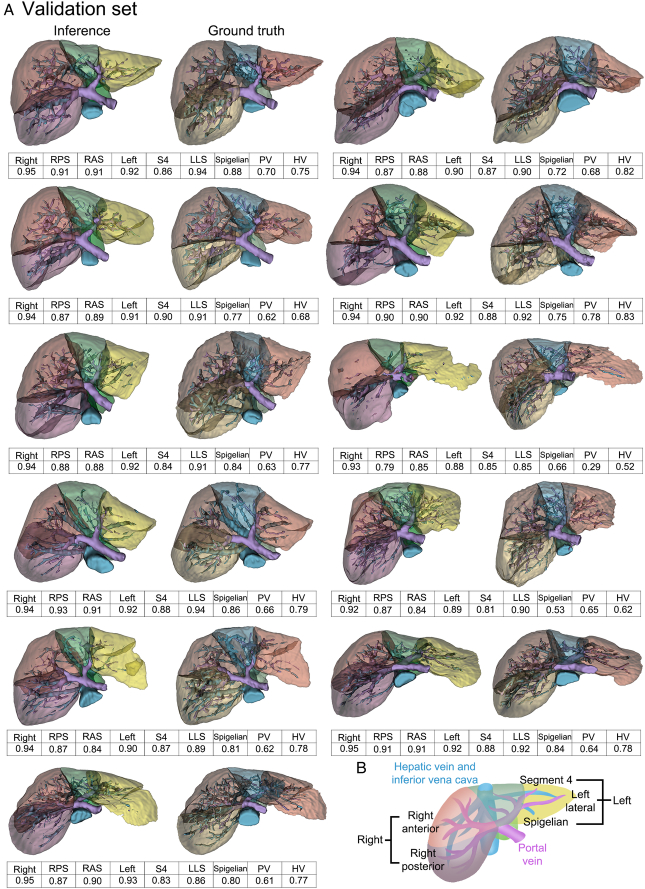
3D reconstruction and segmentation performance in liver parenchyma and vascular structures. (A) This figure illustrates side-by-side 3D reconstructed images of liver parenchyma and vascular structures, comparing the deep learning model’s inference (left column) with the manually segmented ground truth (right column). Each row represents a different patient from the validation set. The models demonstrate segmentation of the right posterior section (RPS), right anterior section (RAS), left medial section (LMS), left lateral section (LLS), Spigelian lobe (SL), portal vein (PV), and hepatic vein (HV). Accompanying dice similarity coefficient (DSC) scores are provided below each structure, indicating the high accuracy of the model compared to the ground truth. (B) This provides a schematic representation of the segmented liver anatomy, including hepatic and portal veins, clarifying the orientation and location of each liver section and vascular structure as identified in the 3D models.

The autosegmentation capabilities of the proposed DL model for parenchyma, PV, and HV are illustrated in in Figure 1, showing 3D reconstructed structures. The DSC for the liver parenchyma yielded a score of 0.94±0.01 for the right lobe and 0.91±0.02 for the left lobe. For individual sections, the DSC scores were as follows: RPS 0.88±0.04, RAS 0.88±0.03, LMS 0.86±0.03, LLS 0.90±0.03, and SL 0.77±0.10. The PV and HV had mean DSC scores of 0.63±0.12 and 0.74±0.09, respectively (Table 2).

**Table 2:** Dice similarity coefficient (DSC) for liver parenchyma, PV, and HV for the validation set

Case number	Right lobe	Right posterior section	Right anterior section	Left lobe	Left medical section	Left lateral section	Spigelian lobe	Portal vein	Hepatic vein
1	0.95	0.91	0.91	0.92	0.86	0.94	0.88	0.70	0.75
2	0.94	0.87	0.88	0.90	0.87	0.90	0.72	0.68	0.82
3	0.94	0.87	0.89	0.91	0.90	0.91	0.77	0.62	0.68
4	0.94	0.90	0.90	0.92	0.88	0.92	0.75	0.78	0.83
5	0.94	0.88	0.88	0.92	0.84	0.91	0.84	0.63	0.77
6	0.93	0.79	0.85	0.88	0.85	0.85	0.66	0.29	0.54
7	0.94	0.93	0.91	0.92	0.88	0.94	0.86	0.66	0.82
8	0.92	0.87	0.84	0.89	0.81	0.90	0.53	0.65	0.62
9	0.94	0.87	0.84	0.90	0.87	0.89	0.81	0.62	0.78
10	0.95	0.91	0.91	0.92	0.88	0.92	0.84	0.64	0.78
11	0.95	0.87	0.90	0.93	0.83	0.86	0.80	0.61	0.77
Mean	0.94±0.01	0.88±0.04	0.88±0.03	0.91±0.02	0.86±0.03	0.90±0.03	0.77±0.10	0.63±0.12	0.74±0.09

### Volumetric comparison between surgeon’s manual and 3D model-based method

**Figure 2:**
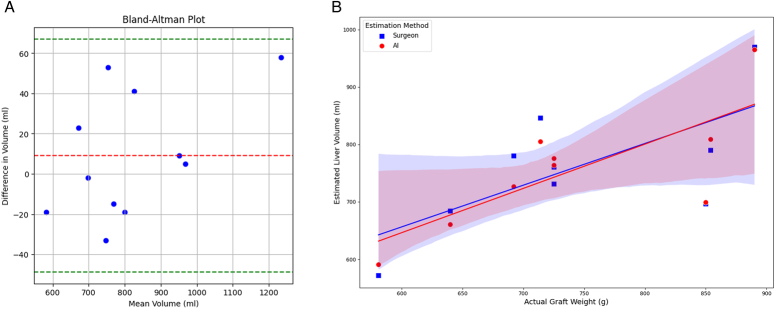
Bland–Altman plot and correlation analysis for volumetric assessment accuracy. (A) Bland–Altman plot assessing the agreement between the liver volume estimates from the deep learning (DL) model and those from the surgeon’s manual measurements. The *y*-axis represents the difference in volume between the two methods, while the *x*-axis shows the average volume estimated by both. The dashed red line indicates the mean difference, and the dashed green lines indicate the upper and lower limits of agreement. (B) This presents a correlation analysis between the actual graft weights and estimated liver volumes. Here, the *y*-axis depicts the estimated liver volume by each method, the *x*-axis represents the actual graft weight, with the surgeon’s estimates shown in blue square and the AI estimates in red circle. The lines of best fit for each method are drawn to illustrate the relationship, with the shaded area representing the confidence interval for each line.

A comparison of graft volume estimates derived from the surgeon’s manual measurements and the DL model for the 11 test set donors is presented in Table 3. Bland–Altman analysis indicated a mean discrepancy of 9.18 ml between the two methods, with the majority of estimates falling within the limits of agreement, which spanned from −48.73 ml to 67.09 ml (Fig. 2A).

**Table 3:** Comparison of Body weight, actual graft weight, and liver volume estimates

Case number	Body weight at the time of CTA (kg)	Body weight at the time of donation (kg)	Absolute weight difference between CTA and operation (kg)	Actual graft weight (g)	Estimated liver volume (ml) by surgeon	Estimated liver volume (ml) by AI
1	64.7	64.8	0.1	692	780	727
2	51.5	49.5	2.0	581	572	591
3	67	69.4	2.4	854	790	809
4	61	62.1	1.1	714	846	805
5	92	N/A	N/A	N/A	956	947
6	55.1	55.5	0.4	725	731	764
7	64.1	62.8	1.3	640	684	661
8	89.1	81.8	7.3	950	1263	1205
9	67.9	68.5	0.6	850	697	699
10	72.9	76.6	3.7	890	970	965
11	63	66.6	3.6	725	761	776

CTA, Computed Tomography Angiography; AI, Artificial Intelligence.

### Correlation between estimated liver volume and actual graft weight

From the 11 donors in the test set, 10 proceeded with donation surgery. The actual graft weights for these donors are documented in Table 3. We calculated the correlation between actual graft weights and liver volumes estimated by both the surgeon and the DL model. One donor with a significant weight loss from the initial (89.1 kg) to actual surgery date (81.8 kg) was excluded for this analysis, while for the remaining nine donors, the difference in body weight from CTA to operation was 1.69±1.33 kg. The surgeon’s estimates had an R-squared value of 0.68 (*P*=0.044), while the DL model demonstrated a higher correlation with an R-squared value of 0.76 (*P*=0.018), suggesting that the DL model’s volume predictions have a stronger association with the actual graft weights (Fig. 2B).

## Discussion

This study aimed to validate the capability of a DL model to automatically segment the parenchyma and vascular structures from CTA of liver donors, and to evaluate the liver volume derived from these segmentations. Our findings indicate that the proposed DL model achieved high concordance with the 3D models manually crafted by biomedical engineers, as evidenced by achieving DSC of 0.94±0.01 for the right lobe and 0.91±0.02 for the left lobe. Furthermore, the liver volume estimations calculated by the DL model had a mean discrepancy of only 9.18 ml when compared to the surgeons’ manual estimates, indicating substantial agreement. Notably, the model’s estimations correlated more closely with the actual graft weights than the surgeons’ assessments did, as reflected by R-squared values of 0.76 for the DL model compared to 0.68 for the surgeon. These findings underscore the potential of DL models to enhance preoperative planning and suggest that they may be a reliable adjunct to traditional methods for estimating liver volume in living donor liver transplantation.

The performance of our DL model in segmenting liver parenchyma for donor surgery was comparable to the previous studies. Koitka *et al*.^13^’s research utilizing a U-Net architecture reported higher performance in measurements of the entire liver, right lobe, and left lobe, with DSC of 0.97±0.01, 0.96±0.01, and 0.92±0.02, respectively. While, Jeong *et al*.^14^’s Deep 3D attention CLSTM U-Net-based model, which incorporated an attention mechanism, reported DSCs of 0.87±0.07 and 0.80±0.08 for the right and left lobes, respectively. While these studies reported promising results, they were limited to segmenting only the right and left lobes of the liver. Our study extends beyond this scope by including critical segments such as the RPS and LLS, which are often used in liver donation surgeries^15–17^. This expansion not only showcases the model’s robustness but also aligns more closely with the diverse graft requirements encountered in clinical practice, thus enhancing the model’s utility and relevance in a real-world clinical setting. The novelty of our model is that the model can be utilized in liver resection beyond donor surgery. Our model included segmentation of the portal and HV, which are important structures for partial hepatectomy. By the intrahepatic vascular structures, the surgeon can design the surgical plane 3-dimensionally. The reason why we divided the liver parenchyma into four sections and SL is that we intended this model to be used for liver surgery other than donor hepatectomy. The volumetric information is important when major hepatectomy is performed while its importance is faded during minor liver resection. We concluded that the five territory is the optimal information that we can provide to the users. The five sectional territories can give diverse options for surgeons. The combinations for major hepatectomy include 10 combinations which are, right posterior sectionectomy, right anterior sectionectomy, right hepatectomy, right trisectionectomy, central hepatectomy, left trisectionectomy, left hepatectomy, segmentectomy of segment IV, left lateral sectionectomy, and left hepatectomy with caudate lobectomy.

While SL only has small volume compared to the whole liver, it is important when left hepatectomy without caudate lobectomy is performed. During donor left hepatectomy, the SL can be both included and excluded from the liver graft. As presented in Table 1, the mean volume of SL was 31.7±27.2 ml, which can be applied during volume assessment of left hepatectomy.

This study has confirmed that the volumetric predictions made by the DL model are in close agreement with those made by surgeons, as evidenced by Bland–Altman plots, suggesting the DL model’s predictions are within statistically acceptable ranges. Moreover, the DL model demonstrated a higher correlation with the actual graft weight compared to surgeons’ estimates, with scatter plots showing AI predictions nearer to the trendline, indicating reduced absolute error. These findings reveal that while conventional preoperative measurements by surgeons can entail interobserver variability due to interpersonal and intrapersonal discrepancies^18^, the DL model offers consistent and stable results. The accuracy of the DL model to human surgeons coupled with its consistency presents a significant opportunity to automate this estimation process. This could potentially eliminate the time investment required by surgeons in volume estimation, thereby offering a substantial efficiency gain^11,12^.

This experimental study showed promising results in segmenting liver parenchyma and vascular structures. However, a notable limitation is the question of its generalizability across different patient populations and varying CT acquisition protocols. As the model was developed using data from a single institution adhering to a specific CT angiography acquisition protocol, there is a need for further research to validate its applicability and accuracy in broader clinical settings. Future studies should focus on training the model with diverse datasets from multiple institutions and testing it with different imaging protocols to practicality ensure its robustness and reliability for widespread clinical adoption. This step is crucial to move from experimental results to a universally applicable clinical tool, enhancing the model’s impact on patient care and surgical planning in liver transplantation. Nevertheless, the novelty of developing this DL model comes from the actual practicality. The preciseness of this model showed that it can be used for living liver donor screening, which can minimize human labor. The variety of this segmentation components can be utilized for surgeons who are planning liver resection based on CT scan. While our data presented the initial result of the model, it will be updated with better performance extending its role as an actual artificial intelligence medical software product in the future.

## Conclusion

Our study confirms that DL model demonstrates potential as a reliable tool for enhancing preoperative planning in liver transplantation, offering consistency and efficiency in volumetric assessment. Further validation is required to establish its generalizability across various clinical settings and imaging protocols.

## Ethical approval

This retrospective study was approved by the institutional review board of Samsung Medical Center (SMC 2024-03-107).

## Consent

The need for informed consent was waived by the IRB due to the retrospective nature of the study.

## Source of funding

This study was supported by Future Medicine 2030 Project of the Samsung Medical Center [SMX1240801, SMX 1230771], Ministry of Science and ICT (RS-2023-00222838), and Korea Health Technology R&D project through the Korea Health Industry Development Institute (KHIDI), funded by the Ministry of Health & Welfare (HI23C038700).

## Author contribution

J.R. and W.K.J.: study design; N.O., J.R., G.-S.C., J.M.K., and J.-W.J.: data collection; N.O., J.-H.K.: data analysis; N.O., J.-H.K., J.R., and W.K.J.: writing the paper.

## Conflicts of interest disclosure

All authors have no conflict of interest.

## Research registration unique identifying number (UIN)

Not applicable.

## Guarantor

Jinsoo Rhu and Woo Kyoung Jeong.

## Data availability statement

Data analyzed during the study are available from the corresponding author by request. Data generated or analyzed during the study are available from the corresponding author by request.

## Provenance and peer review

Not applicable.

## References

[1] Hiroshige S Shimada M Harada N . Accurate preoperative estimation of liver-graft volumetry using three-dimensional computed tomography. Transplantation 2003;75:1561–1564. 12792515 10.1097/01.TP.0000053755.08825.12

[2] Radtke A Sotiropoulos GC Nadalin S . Preoperative volume prediction in adult living donor liver transplantation: how much can we rely on it? Am J Transplant 2007;7:672–679. 17229068 10.1111/j.1600-6143.2006.01656.x

[3] Kim SH Kim KH Cho HD . Donor safety of remnant liver volumes of less than 30% in living donor liver transplantation: a systematic review and meta-analysis. Clin Transplant 2023;37:e15080. 37529969 10.1111/ctr.15080

[4] Taner CB Dayangac M Akin B . Donor safety and remnant liver volume in living donor liver transplantation. Liver Transpl 2008;14:1174–1179. 18668669 10.1002/lt.21562

[5] Kim DG Hwang S Kim JM . Outcomes and risk factors for liver transplantation using graft-to-recipient weight ratio less than 0.8 graft from living donors: multicentric cohort study. Ann Surg 2024;279:1018–1024. 37753651 10.1097/SLA.0000000000006104

[6] Matsushima H Soyama A Hara T . Outcomes of living donor liver transplant recipients receiving grafts with the graft-to-recipient weight ratio less than 0.6%: A matched pair analysis. Liver Transpl 2024;30:519–529. 37788305 10.1097/LVT.0000000000000276

[7] Schiano TD Bodian C Schwartz ME . Accuracy and significance of computed tomographic scan assessment of hepatic volume in patients undergoing liver transplantation. Transplantation 2000;69:545–550. 10708109 10.1097/00007890-200002270-00014

[8] Lim MC Tan CH Cai J . CT volumetry of the liver: where does it stand in clinical practice? Clinical radiology 2014;69:887–895. 24824973 10.1016/j.crad.2013.12.021

[9] Karlo C Reiner CS Stolzmann P . CT- and MRI-based volumetry of resected liver specimen: comparison to intraoperative volume and weight measurements and calculation of conversion factors. Eur J Radiol 2010;75:e107–e111. 19782490 10.1016/j.ejrad.2009.09.005

[10] Rhu J Choi GS Kim MS . Image guidance using two-dimensional illustrations and three-dimensional modeling of donor anatomy during living donor hepatectomy. Clin Transplant 2021;35:e14164. 33222255 10.1111/ctr.14164

[11] Oh N Kim JH Rhu J . Automated 3D liver segmentation from hepatobiliary phase MRI for enhanced preoperative planning. Sci Rep 2023;13:17605. 37848662 10.1038/s41598-023-44736-w PMC10582008

[12] Oh N Kim JH Rhu J . 3D auto-segmentation of biliary structure of living liver donors using magnetic resonance cholangiopancreatography for enhanced preoperative planning. Int J Surg 2024;110:1975–1982. 38668656 10.1097/JS9.0000000000001067 PMC11020049

[13] Koitka S Gudlin P Theysohn JM . Fully automated preoperative liver volumetry incorporating the anatomical location of the central hepatic vein. Sci Rep 2022;12:16479. 36183002 10.1038/s41598-022-20778-4 PMC9526715

[14] Jeong JG Choi S Kim YJ . Deep 3D attention CLSTM U-Net based automated liver segmentation and volumetry for the liver transplantation in abdominal CT volumes. Sci Rep 2022;12:6370. 35430594 10.1038/s41598-022-09978-0 PMC9013385

[15] Cho CW Choi GS Kim KS . Surgical techniques and outcomes of pure laparoscopic donor right posterior sectionectomy for living donor liver transplantation. Liver Transpl 2022;28:325–329. 34314564 10.1002/lt.26244

[16] Yoshizumi T Ikegami T Kimura K . Selection of a right posterior sector graft for living donor liver transplantation. Liver Transpl 2014;20:1089–1096. 24890095 10.1002/lt.23924

[17] Scatton O Katsanos G Boillot O . Pure laparoscopic left lateral sectionectomy in living donors: from innovation to development in France. Ann Surg 2015;261:506–512. 24646560 10.1097/SLA.0000000000000642

[18] Plank B . The’Problem’of Human Label Variation: On Ground Truth in Data, Modeling and Evaluation. arXiv preprint arXiv:2211.02570. https://arxiv.org/abs/2211.02570

